# Biometrics for Industry 4.0: a survey of recent applications

**DOI:** 10.1007/s12652-023-04632-7

**Published:** 2023-05-31

**Authors:** Cascone Lucia, Gao Zhiwei, Nappi Michele

**Affiliations:** 1 University of Salerno, Fisciano, Italy; 2 University of Northumbria, Newcastle upon Tyne, UK

**Keywords:** Workplace health promotion, Smart grid, Industrial internet of things, Cloud computing, Recognition, Security

## Abstract

The Fourth Industrial Revolution, also known as Industry 4.0, represents the rise of digital industrial technology that is propagating at an exponential rate compared to the previous three revolutions. Interoperability is a basis of production, where there is a continuous exchange of information between machines and production units that act autonomously and intelligently. Workers play a central role in making autonomous decisions and using advanced technological tools. It may involve using measures that distinguish individuals, and their behaviours and reactions. Increasing the level of security, allowing only authorized personnel access to designated areas, and promoting worker welfare can have a positive impact on the entire assembly line. Thus, capturing biometric information, with or without individuals’ knowledge, could allow identity verification and monitoring of of their emotional and cognitive states during the daily actions of work life. From the study of the literature, we outline three macro categories in which the principles of Industry 4.0 are merged and the functionalities of biometric systems are exploited: security, health monitoring, and quality work life analysis. In this review, we present an overview of all biometric features used in the context of Industry 4.0 with a focus on their advantages, limitations, and practical use. Attention is also paid to future research directions for which new answers are being explored.

## Introduction

**Fig. 1:**
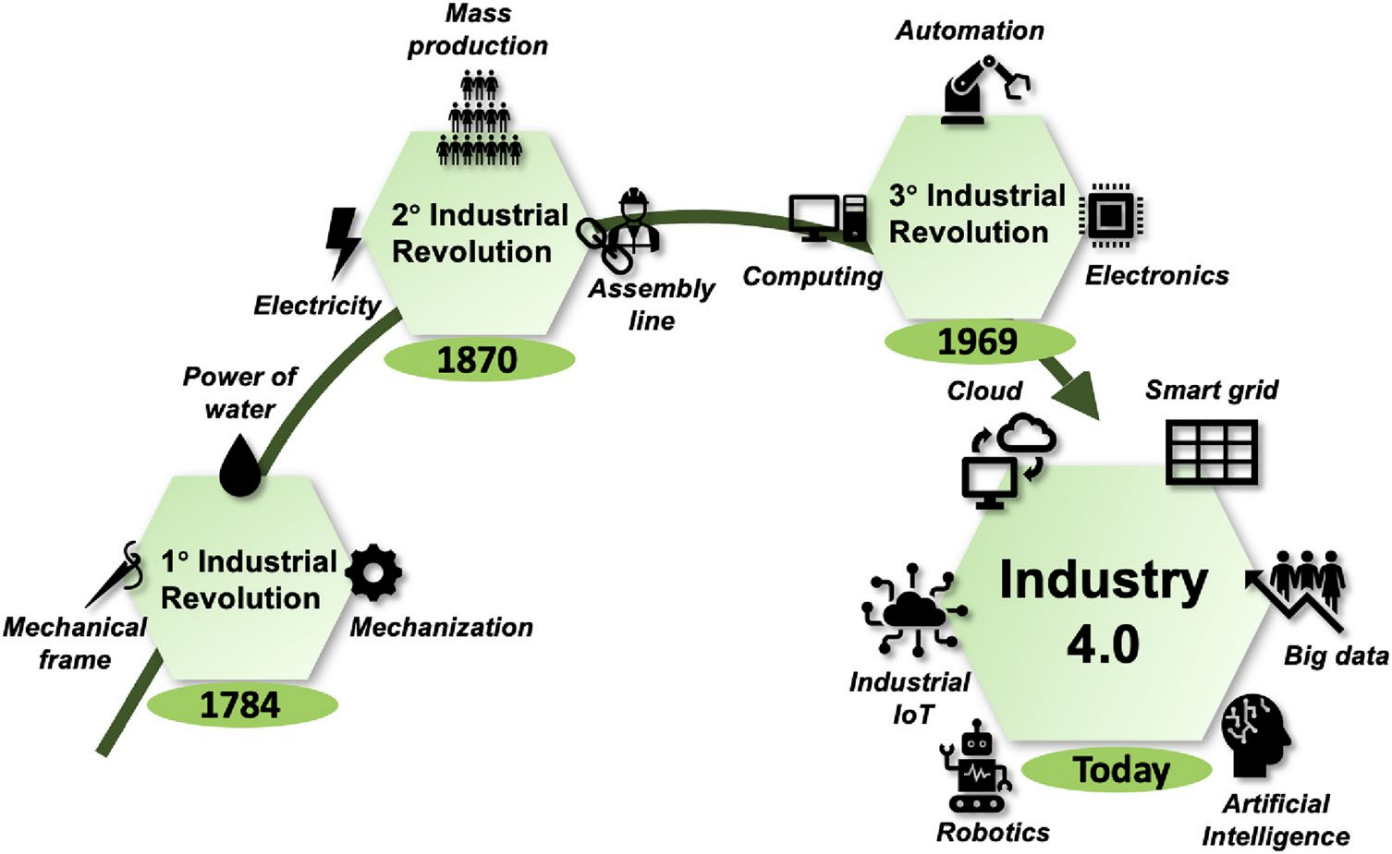
Major technological innovations for the various industrial revolutions, from the first one to the Industry 4.0

Industry 4.0 represents a hot and great relevance topic that individuals, companies and governments alike are approaching to kick-start a new, cutting-edge way of doing industry (Ahmed et al. 2022). This paradigm denotes a complex process that, in contrast to earlier industrial revolutions, does not occur after a specific innovation and/or invention. It exploits and integrates within a business context software and hardware technologies that are already well-known and used in other areas (Fig. 1). The resources available must be managed at a higher level, minimizing the human contribution (Mittal et al. 2018). The goal of this new trend is to have a fully automated production and business management model where machines, people and products are interconnected (Chen et al. 2021). In this way it is possible to achieve a greater impact on production, automation and digitalization of various operations and, finally, greater effectiveness and efficiency of the various involved processes (Ahmed Khan et al. 2021; Kamble et al. 2018).

The Industry 4.0 paradigm involves various technologies and concepts such as big data processing (Ruiz et al. 2021), robotics (Nie and Chen 2021), cloud computing (Dohare et al. 2022), artificial intelligence (Yuan and Cai 2022), industrial internet of things (IIoT) (Anajemba et al. 2022) and many others (Ebrahimpour et al. 2022). When these industrial applications are integrated in the same system, it is possible to observe a bridge between the physical and digital worlds and the full potential of Industry 4.0.

**Table 1:** Comparison of the biometrics reported in the work with respect to seven properties whose values were assigned based on the findings of the literature review

Biometrics	Acceptability	Collectability	Uniqueness	Performance	Permanance	Universality	Vulnerability	Industry 4.0 applications	Publications
*Physical biometrics*									
BMI	M	M	L	L	L	H	H	WHP	Äikäs et al. 2020; Rosemberg et al. 2019; Reif et al. 2020
Face	H	H	M	M	M	H	H	Security	Wati et al. 2021; Alkeem et al. 2021
Fingerprint	H	M	H	H	H	H	M	Security	Golec et al. 2022; Alkeem et al. 2021
Hand vein	M	M	M	M	M	M	L	Security	Zhao et al. 2021
Iris	L	M	H	M	M	H	L	Security	Ma et al. 2020
*Physiological biometrics*									
Blood pressure	M	M	L	L	M	H	L	WHP-QWL	Yu et al. 2017; Song and Baicker 2019; Äikäs et al. 2020; Rosemberg et al. 2019; Girardi et al. 2021; Reif et al. 2020
ECG	H	M	M	M	M	H	L	Security	Alkeem et al. 2021
EEG	L	L	M	M	L	H	L	WHP-QWL-Security	Bacevice and Ducao 2021; Ramírez-Moreno et al. 2021; Libert and Van Hulle 2021
Galvanic skin response	M	M	L	L	L	H	H	WHP-QWL	Girardi et al. 2021; Soto et al. 2021; Ramírez-Moreno et al. 2021
Heart rate	H	H	L	L	L	H	L	WHP-QWL	Bacevice and Ducao 2021; Züger et al. 2018; Girardi et al. 2021; Concheiro-Moscoso et al. 2021; Soto et al. 2021; Äikäs et al. 2020Rosemberg et al. 2019
Respiration rate	H	M	L	L	L	H	H	WHP-QWL	Soto et al. 2021
Skin temperature	H	H	L	L	L	H	H	WHP-QWL	Soto et al. 2021; Ramírez-Moreno et al. 2021
*Behavioral biometrics*									
Eye movements	H	M	L	M	L	H	M	Security	Ma et al. 2020
Facial dynamics	H	M	L	M	L	H	M	Security	Castiglione et al. 2020
Keystroke	H	M	L	L	L	L	M	WHP	Ulinskas et al. 2018
Signature	H	H	L	L	L	L	H	Security	Bhowal et al. 2022
Speech signal	H	M	L	L	L	M	H	Security	Srivastava et al. 2019
Posture patterns	H	M	L	L	L	M	H	Security	Kaczmarek et al. 2018
Pressure distributions	H	M	L	L	L	H	L	WHP	Zhang et al. 2021

*H* high, *M* medium, *L* low

With Industry 4.0, even the way of understanding the worker has changed. Specific technical skills and abilities (hard skills), will give way to more transversal, generic and less sectoral skills. Mental labor will play a more significant role, and worker responsibility will increase. Job stress can cause demotivation, lack of sleep and consequent fatigue. Employees in these situations are not only prone to illness and unable to fully contribute to job duties, but also risk being in situations that are dangerous to themselves and others. Therefore, the need to have greater security in access controls to sensitive areas, to verify the performance of work, to intercept any situations of fatigue or stress that may affect safety and, finally, the desire to promote a satisfactory working life are just some of the aspects that the industry of the future must be able to manage. Through the analysis of specific biometric characteristics, it is possible to try to give an answer to each of these needs, intercepting the various information necessary to capture people’s reactions, emotions and identity. The acquisition of biometric information whenever the subject is aware of it or not, allows to extract latent knowledge and monitor the subject during the actions of working life. Biometrics exploits measurable physiological, physical and behavioural traits of humans for purposes of recognition and analysis (Kumari and K.R. 2019).

Physiological traits are correlated with bodily functions and processes, such as heart rate, blood pressure, respiratory rate, hormone levels, and brain activity (Wasimuddin et al. 2020). These traits can be measured using various techniques, including electrocardiography, electroencephalography, magnetic resonance imaging, and blood tests. They are particularly interesting because they can provide information on an individual’s health, fitness, and emotional states. Physiological biometrics have the enormous advantage of being practically impossible to replicate or counterfeit, making them a highly secure option. The main limitations associated with them are undoubtedly the difficulty of acquiring this data and the sensitivity of sensors to environmental conditions. Physical biometric traits are observable characteristics of an individual’s body, such as height, hair color, face, and fingerprints (Rattani and Derakhshani 2018b). These traits are determined by genetics but can be influenced by other factors, such as diseases, injuries, surgeries, and traumas. These events can temporarily or permanently modify some of a person’s physical characteristics. These biometric traits have the advantage of being easily acquired and used, but they also have limits in terms of security, as they can be falsified or imitated. Moreover, it is important to emphasize that these biometrics can still change over time, and their use must be carefully weighed, considering also the implications in terms of privacy and security, in addition to their reliability and stability over time. Behavioral biometric traits are based on observable actions and reactions of an individual, such as keystroke dynamics, gait, signature, and language patterns (Cascone et al. 2020). These biometric traits can be used in situations where it is not possible to acquire a physical or physiological biometric data, but they have the limitation of being influenced by external factors, such as fatigue or stress. Collectively, a detailed analysis of physiological, physical, and behavioral traits can provide valuable information on an individual’s health, fitness, and emotional states, as well as their identity, personality, cognition, and social behaviors. In general, seven characteristics describe biometric traits: acceptability, collectability, uniqueness, performance, permanence, universality, and vulnerability. In Table 1, highlighting the group they belong to, we report on the basis of these properties a comparison, between the biometric characteristics that will later be the focus of attention in this paper.

Therefore, despite the obvious potential benefits of integrating biometric techniques in the context of Industry 4.0, their adoption is still limited. Although the state of the art suggests various research directions, there is still no standard, focused study that fully integrates and shows the potential of these two paradigms. For example, in studies such as of De Keyser et al. (2021), more general considerations and analyses of biometric applications in various and generic business sectors are reported, and, mostly, more from the perspective of consumers than providers as in this survey. For this reason, in this article we examine for the first time, to the best of our knowledge, the Industry 4.0 paradigm by analyzing its principles and the main technologies involved, in relation to physical, physiological and behavioral biometrics. This information, if integrated into this new industrial landscape, could provide useful information about the identity of the worker, the cognitive aspects in which he or she is and how he or she perceives the work environment. From our review of the literature, we have identified three macro-areas where biometrics and the new work paradigm converge: security, workplace health promotion (WHP) and quality work life (QWL). In Table 1 we highlight for each biometric trait that will be analysed below, the area in which it was exploited.

Our contributions can therefore be summarized as follows:

- Collection, classification and synthesis of works in the context of Industry 4.0 along with their main aspects into an industrial context-specific taxonomy. Specifically, they were grouped with respect to three areas of study: security, health monitoring, and quality work life analysis. Specific focus on the biometric characteristics used in each paper analyzed is provided.
- Identification of the main challenges, limitations, implications, opportunities and future research directions for which new responses are being explored to transform current manufacturing in the Industry 4.0 paradigm.

**Fig. 2:**
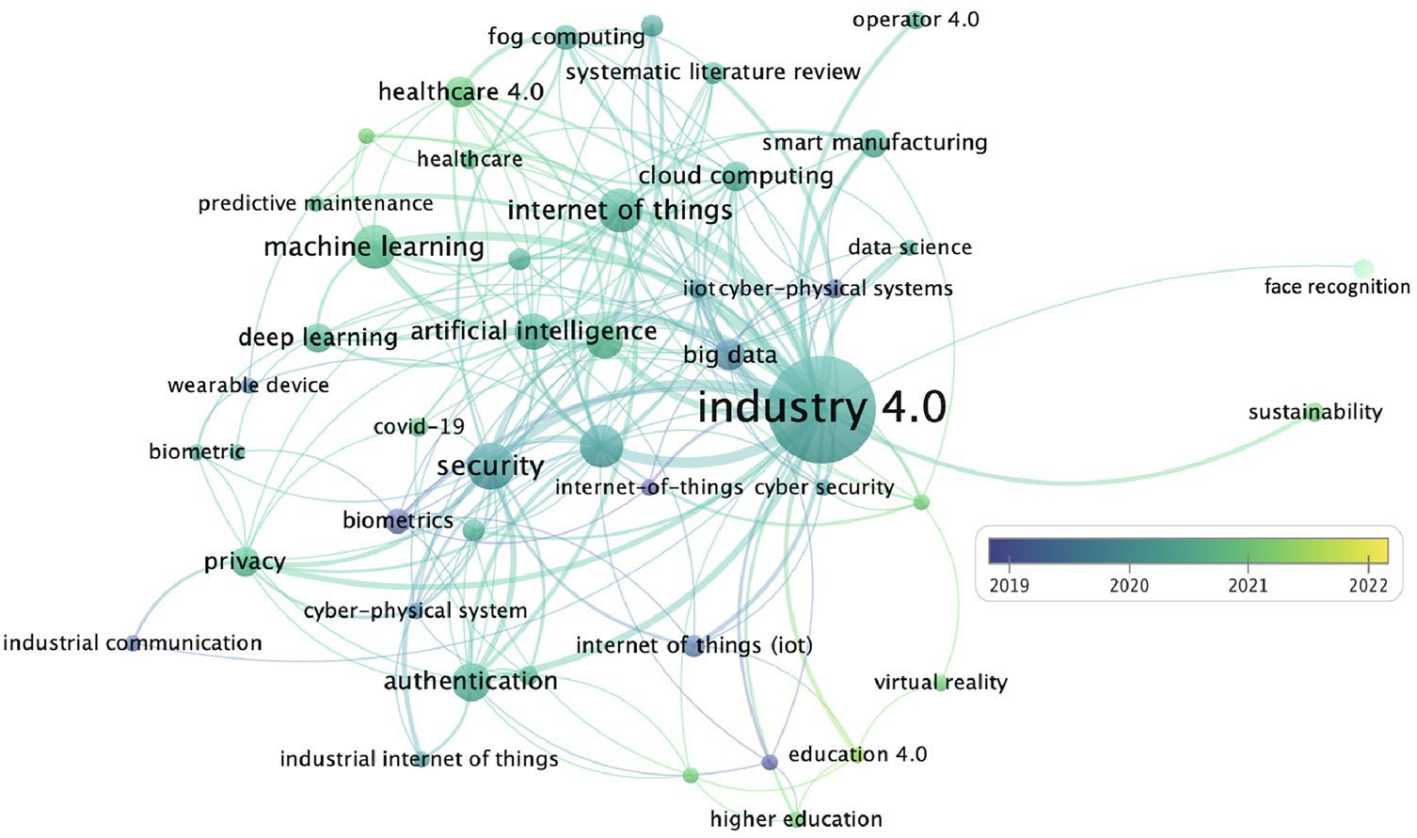
The keywords of the most recent research articles on Industry 4.0 and biometrics. The node’s size represents the number of occurrences, while its color indicates the publication year. Graph produced with the VOSviewer software

**Fig. 3:**
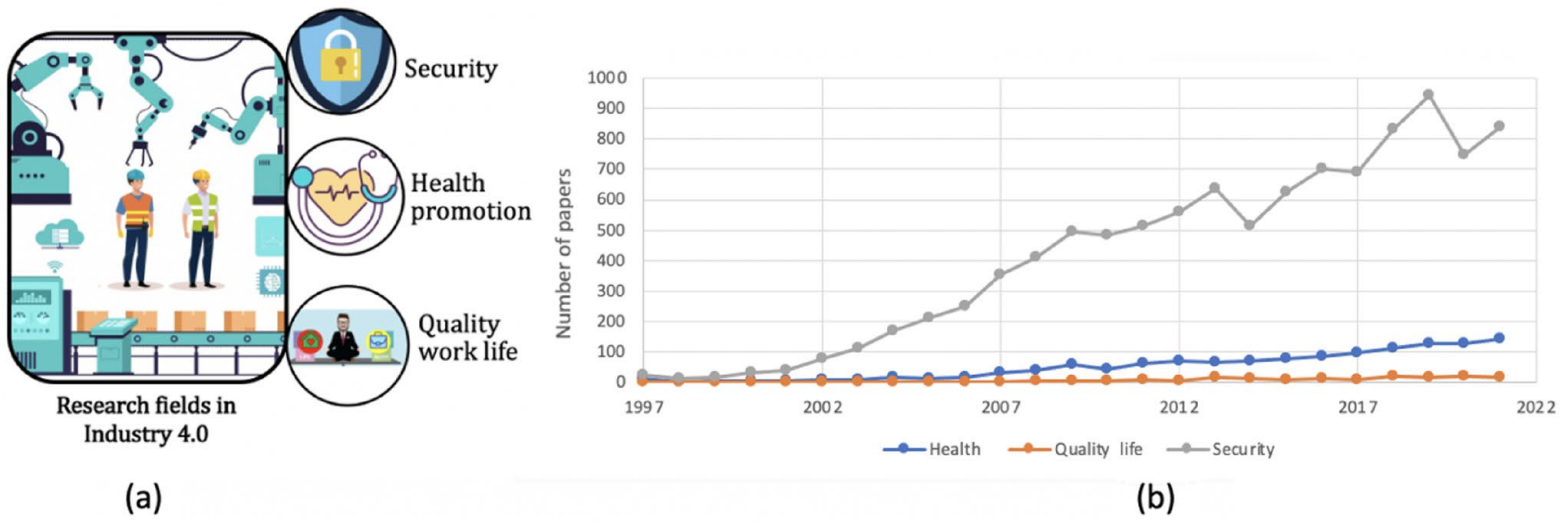
**a** The main recent research fields in Industry 4.0. **b** Distribution over the years of biometric works on security issues, quality of life analysis, and health monitoring

**Fig. 4:**
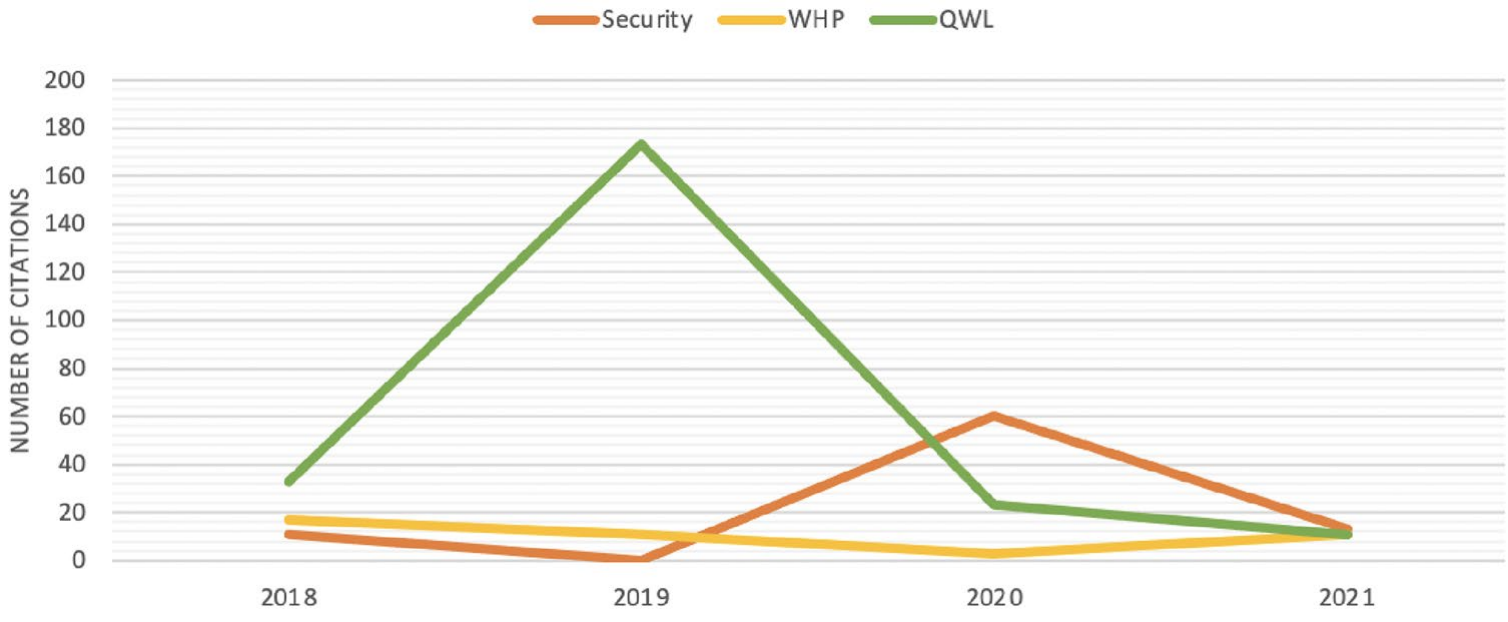
Cumulative number of citations over the years for reported papers with respect to the 3 macro areas of security, health monitoring, quality work life

## Methodology

Fig. 2, created using VOSviewer,^1^ is a network visualization that enables the analysis and graphical representation of the relationships between the keywords within all the documents shown by Scopus using “Industry 4.0” and “biometrics” as keywords. In this network map, keywords are represented as nodes, while the relationships between them are represented as lines. The size of the node represents the number of occurrences, while its color indicates the year of publication. Inspired by this graph and the literature present, we have identified three different domains in which to analyze the Industry 4.0 paradigm in relation to the use of biometrics: security, health monitoring, and biometrics for a quality work life (Fig. 3a). Figure 3b shows a graph that displays the trend of studying these topics in general in biometric documents over the years. Can Industry 4.0 actually benefit from the introduction of biometric systems? Which area is the most investigated, and which one has the most potential? These were the first questions we asked ourselves. After a thorough investigation of the literature, gaps in research and studies applied in real industrial contexts emerged, which we will better highlight below. To study the literature and select the documents, we consulted the most recent articles from 2017 to 2022 identified through Scopus,^2^ ACM DL,^3^ and IEEEXplore,^4^ mainly selecting those from high-level journals and conferences, taking into account the SCImago journal rank^5^ indicator for journals and the GII-GRIN-SCIE (GGS) Conference Rating service^6^ for conferences. Among the keywords used are “biometrics,” “Industry 4.0” or “Industry” or “workplace” or “workers,” and then, in turn, the words associated with the three macro-areas identified, such as “security,” “health,” “quality work life” with their subdomains such as “identification,” “authentication,” “stress,” “awakeness,” “fatigue,” “wellness and productivity,” etc. Due to the specific requirements of the research, in fact, it was decided to include articles that did not have an explicit focus on the Industry 4.0 topic in question. Therefore, the literature review also focused on those articles that dealt with subdomains of Industry 4.0 that, however, have applications in the business and commercial world more generally. Figure 4 shows the cumulative citations obtained from the works selected in this investigation grouped according to the three categories of membership. From the graph, it emerges that the theme related to the analysis of quality work life has been particularly trendy and relevant in recent years. Given the obvious benefits that can be derived from it, there is also growing interest from those involved in health monitoring. Finally, the security area confirms itself as a fundamental aspect to study, on which much attention and curiosity is focused.

**Table 2:** Summary table for the area of security. Each research paper is reported according to the biometrics analyzed, the data sample used, the salient strategies, and the most interesting results obtained

Paper	Aim	Biometrics	Dataset	Method	Performance
Ma et al. (2020)	Authentication	Iris eye movement	Private dataset: 8 subjects	Eye-movement trajectory for freshness verification $\cdot$ ML algorithms applied to iris features	Accuracy: 89.67%
Wati et al. (2021)	Authentication	Face	Private dataset: 40 subjects	Viola-Jones for face detection Gabor Wavelet for feature extraction Template matching	*Group* Accuracy: 88% Recall: 75% Precision: 97% *Individual* Accuracy: 75% Recall: 64% Precision: 88%
Zhao et al. (2021)	Authentication	Finger vein	Public datasets: SDUMLA-FV	Analysis of features from local and global perspectives	*SDUMLA-FV* Accuracy: 98.90% *FV-USM* Accuracy: 97.43%
Golec et al. (2022)	Authentication	Fingerprint	Public dataset: FVC-2004 DB3	Information extraction on minutiae Encryption methods	EER: 30% Accuracy: 70%
Bhowal et al. (2022)	Authentication	Signature	Public datasets: SVC 2004 MCYT-100	Physical, frequency-based, and statistical feature extraction A two-tier ensemble approach for classification	*SVC 2004* Accuracy: 98.43% EER: 2.20 GAR@0.01FAR: 94.50% *MCYT-100* Accuracy: 97.87% EER: 2.84 GAR@0.01FAR: 92.90%
Libert and Van Hulle (2021)	Authentication	EEG	Private dataset: 15 subjects	STFT for feature extraction LSTM-based network with bootstrap aggregating	*Performed motor task* Accuracy: 92.6% FAR: 2.5% FRR: 5% *Imagined motor task* Accuracy: 92,5% FAR: 2.6% FRR: 4.9% *Combined tasks* Accuracy: 93% FAR: 1.9% FRR: 5,1%
Kaczmarek et al. (2018)	De-authentication Identification	Posture Patterns	Private dataset: 30 subjects	ML algorithms applied to time series of the force on the sensors	True positive rate: 91.0% False positive rate: 0,33% False negative rate: 8.68% *Multiple sessions* True positive rate: 22%, False positive rate: 5.2%, False negative rate: 72.7%
Castiglione et al. (2020)	Identification Authentication	Facial dynamics	Private dataset: 48 subjects	Local spatial–temporal descriptor for features Deep feedforward network for classification	*Identification* Accuracy: 98.2% *Authentication* Accuracy: 99.49% EER: 0.05%
Srivastava et al. (2019)	Identification	Voice	Private datasets: 10 subjects	LPC and MFCC feature extraction technique GMM classifier	*Isolated Hindi digit dataset* Accuracy: 96.49% *Hindi sentence dataset* Accuracy: 94.97%
Alkeem et al. (2021)	Identification Gender recognition Authentication	Face Fingerprint ECG	Private virtual dataset of 58 subjects based on three different public datasets	ResNet50 for face and fingerprint feature extraction CNN for ECG feature extraction Multimodal approach: feature and score fusions	*Identification* Feature fusion accuracy*: 98.97% Score fusion accuracy*: 98.95% *Gender* Feature fusion accuracy*: 96.55% Score fusion accuracy*: 99.42% * Results of when noise was added to all modalities *Authentication* Accuracy: 100% FAR: 0%

## Security

Traditional password and pin-based recognition methods no longer provide adequate security for accessing sensitive information. The simplicity of passwords and pins makes them very easy to hack and when they become more complex they become difficult to remember, making password management tedious. In this scenario, biometric systems are now an established technology in the security industry, increasingly being used for identity control. Although some biometric systems are harder to breach than others, no security system is impenetrable. There are potential attacks that could target these systems and make them vulnerable. Private information is then exposed to danger with the consequent privacy problems. Security systems for user recognition can be divided into two groups with two different purposes, namely authentication and identification. In the first case, we evaluate whether the user is authorized or not through a one-to-one verification process, while in the second case we recognize the subject who is through a one-to-many verification (Horng et al. 2021). Below we report the most recent works dealing with identification and/or authentication within an Industry 4.0 or work context, with a focus in particular on the biometric features analyzed. Table 2 contains the most important details regarding the papers analysed in this section.

### Authentication

#### Periocular area

With the rise of the Industry 4.0 paradigm, security in smart grid communications between operator and control center has also received special consideration (Rattani and Derakhshani 2018a). Remote network-based attacks can be a threat to smart grid technology, as they can secretly intercept the communication traffic between two parties and even alter it (Yin et al. 2019). For this reason, the use of biometric authentication could be a strong first defensive strategy to prevent such cyber-attacks both when they result in a threat in terms of either privacy or access policies.

To do this, in Ma et al. (2020) the authors proposed an authentication system based on eye movement and iris recognition where for the former biometrics the trajectory was recorded and for the latter instead randomly selected an image. The implementation of this multi-biometric system, with the integration of eye movements, aimed to fight the vulnerability of those systems that used the single static image of the iris. In fact, eye movements were integrated to achieve freshness verification. This system can be easily implemented on a smartphone as it does not require any special hardware requirements other than a high-resolution camera. Moreover, by informally analyzing the security level and using Burrows–Abadi–Needham logic (BAN logic), it has been shown to be able to withstand various known attacks and thus provide a good level of security. With a machine learning (ML) strategy, the accuracy achieved on an 8-subject sample that has been analysed reached approximately 90%. The dataset has not been released.

#### Face

Automated attendance systems, based on biometric data, not only allow for faster checking operations but also help prevent the malpractice of identity exchanges. The use of faces as biometrics is among the most productive image processing applications and plays a central role in the technical field. The face is certainly a biometric that records essential information for the characterisation of a person (Morosan 2020).

In Wati et al. (2021) the authors exploited this trait to create a system that authorized access only to those employees recognized thanks to their face. The hardware needed for such a system is the essential one, i.e., there is no need to buy any particular support devices just a laptop with a webcam that can also be already integrated. Two different strategies were implemented for attendance recording. The first one dealt with the recording of an individual face while the second one coped with a group face when, several faces (five) were captured simultaneously, and then each one was extracted and recognized. This study achieved an accuracy of 75%, a recall of 64%, and a precision of 88% for the recognition of individual attendance. The research achieved 88% accuracy, 75% recall, and 97% precision using simultaneous/group face recognition. Images in the dataset were taken real-time from 40 respondents using a webcam for a total of 1200. The dataset has not been released. In addition to this, after the operations of recording attendance, the system can be made even more interesting and robust by also verifying whether the work shift that the subject is accessing is the right one or not.

#### Vein

With the increasing use of IIoT devices and smart factories, there is a growing risk of cyber-attacks and unauthorized access. Vein biometrics is a safe and accurate way to verify someone’s identity. This lowers the risk of security breaches and makes sure that only authorized people can access sensitive information and equipment. It guarantees a high degree of privacy, as the structure of the finger vein can only be obtained with specialized equipment, making it impossible to steal someone’s finger vein without attracting their attention. This ensures that individuals’ personal information remains secure and protected from unauthorized access. Secondly, finger vein biometrics possess strong anti-cheating abilities, as they require finger activity during imaging. This means that people can’t trick the system by using fake fingers or still images. This lowers the risk of fraud and makes sure that the recognition process is accurate. In order to establish secure access control for authorized personnel to IIoT devices, the authors Zhao et al. (2021) proposed a finger vein recognition system that relied on the characterization of local vein texture features. In particular, local image features were added to those of the whole image. This made it possible to analyze statistical features by processing images from both a local and a global point of view. The experiments were conducted on two public datasets: SDUMLA-FV and FV-USM. The system was found to achieve accuracies exceeding 97% in both databases. However, the quality of images under examination was identified as a significant challenge to the authentication process, as it may significantly impact the accuracy of the system.

#### Fingerprint

In order to guarantee security and privacy between edge devices in the IoT and Industry 4.0, in Golec et al. (2022) the authors proposed a framework for fingerprint-based biometric authentication. In order to enhance security measures and mitigate the risk of hacking attacks, encryption was applied to both the input data and biometric template of the user seeking authentication. To perform a match, the encrypted data was decrypted and compared individually on the server side. Encryption is therefore just another additional security measure. However, it is important to note that, of course, this additional security comes at a cost in terms of time in performance. The encryption and decryption times also depend on the length of the vector being transformed. When implementing this system in tasks that require high responsiveness, this aspect should not be underestimated; processing speed is essential. As usual, access to the system is allowed only if the fingerprint of the person entering matches the stored template, if it does not, then the person cannot enter. The equal error rate EER rate was found to be 30%. Overall system performance records 70% accuracy.

#### Signature

In the era of digitization and Industry 4.0, it is crucial for organizations to replace traditional authentication methods, such as handwritten signatures, with modern digital processes that have equivalent capabilities for document authentication and legal validation  (Kaur and Kumar 2021). The Fourth Industrial Revolution demands the digital transformation of document signatures while leveraging their unique and identifiable characteristics to provide additional security and authentication. For example, in the case of using AI-based software or robots, obtaining a signature from a human employee to validate the performed operations can identify the origin of the instructions (Thumbur et al. 2021). More generally, in the context of Industry 4.0, signatures could be used to track the movement and performance of each product or asset throughout the entire supply chain. However, that of signature forgery is a problem that has always affected all sectors, from education to finance.

Therefore, the goal of the work of Bhowal et al. (2022) was to develop an authentication system for online signature verification that can efficiently identify when the analyzed signature was authentic or counterfeit and cleverly crafted. The approach used was based on two levels. In the first, three different categories of features: physical and frequency- and statistics-based, were extracted and given as input to seven classifiers. The decisions were then evaluated in the second level where a majority voting strategy was implemented to make the final decision, i.e., whether the signature in question was authentic or counterfeit. Two standard datasets, SVC 2004 (Task-II) and MCYT-100, were used to evaluate the system. On the SVC 2004 dataset, the EER and accuracy were 2.20 and 98.43%, while on the MCYT-100 dataset, they were 2.84 and 97.88%, respectively. The GAR@0.01FAR value for the SVC 2004 dataset was 94.50%, while for the MCYT-100 dataset it was 92.90%. The genuine acceptance rate (GAR) is identical to the true acceptance rate and the true positive rate. It is the proportion of authentic samples accepted by a biometric system or algorithm. False acceptance rate (FAR) is the proportion of forged samples accepted as genuine by a biometric system. The value GAR@0.01FAR is derived from the receiver operating characteristic (ROC) curve in which the value of GAR is determined when the FAR is 0.01.

#### Electroencephalography

The expression patterns of spontaneous electrical activity in the brain are not only subject-specific, but are particularly interesting because, not being a visible feature and being involuntary (imitating one’s own mind is impossible), they do not lend themselves to the possibility of easy cloning or coercion (Arsalan and Majid 2021). For these reasons, there are many electroencephalogram (EEG) based identity authentication methods in the literature.

Among them, Libert and Van Hulle (2021) in their work proposed a brainwave authentication system. In particular, the results obtained in this work showed the potential of using this biometric trait to authenticate the identity of a subject in a real-life context (which could also be the work environment) even with only the use of commercial and not particularly accurate hardware (a commercial EEG headset with a dry electrode and chronic recordings). To simulate a real-life environment, sessions were held in the subject’s residence and during each acquisition they were asked to settle into a comfortable position and follow instructions presented on the screen. External interferences were reduced (lights and electronic devices turned off). Recruited 15 healthy subjects, first, 10 trials of the performed motor paradigm were executed, including 5 trials with the left arm and 5 trials with the right arm, all in a random order, and then 10 trials of the imagined motor task were conducted. The screen displayed a clear instruction between the performed and imagined motor paradigms. Short-term Fourier transform (STFT) was used to calculate the spectrogram of each electrode signal, then serving as input for long-term memory networks (LSTM). The authentication accuracy, FAR, and FRR were 92.6%, 2.5%, and 5.0% for the performed motor task; 92.5%, 2.5%, and 4.9% for the imagined motor task; and 93.0%, 1.9%, and 5.0% for the combined tasks, respectively. By conducting additional research and integrating more sophisticated signal functions and decoders, the system’s performance could be enhanced, leading to a greater range of applications in an intelligent work setting.

#### Continuous authentication

**Fig. 5:**
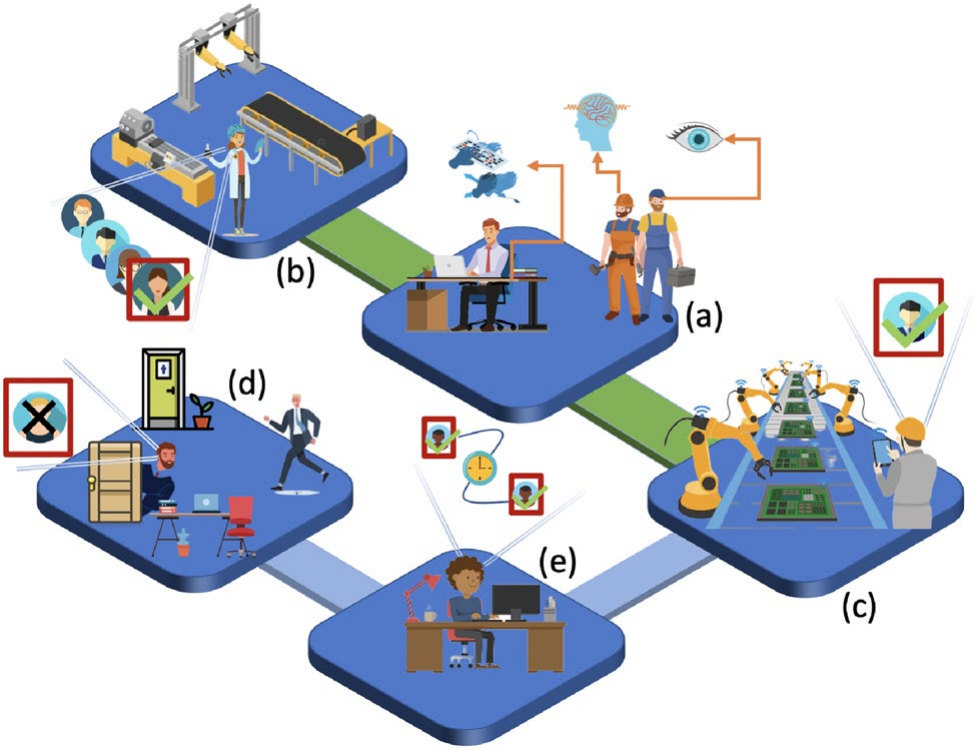
**a** A pool of biometric inputs, physiological, physical and behavioral, can be used in an identification (**b**) and/or authentication (**c**) system. **e** Authentication can be continuous. **d** A particular sub-case of the latter is de-authentication

Authentication is the process of establishing the veracity of an assertion, such as a user’s identity. This can also occur continuously and in real time (Fig.  5e). Traditionally, authenticating a user at login only, or with two factors (second level of security after login), does not eliminate the possibility that, during a certain activity, the user is no longer who was authenticated. To remedy this, continuous authentication is therefore necessary.

It is a strategy that aims to confirm the identity of the individual continuously throughout the activity (Stylios et al. 2021). Verification occurs primarily in the background, without influencing and interrupting the user’s flow of actions. It is often implemented by also integrating behavioral biometric models, building on the user’s natural interactions without constantly interrupting them. In Industry 4.0, there is a need to have real-time communication between cyber-physical systems and workers in charge of various tasks. It is necessary to develop interfaces and services that allow the user to have this interaction in a simple and immediate way, but at the same time it is necessary to ensure a high degree of protection of the data that is given access to, making sure that only authorized personnel can view it.

With the goal of having continuous authentication, in Liang et al. (2020) the authors provided a systematic overview of behavioral biometric features that can be used for this purpose. In particular, they focused their attention in the context of IoT and various IoT devices ranging from wearable sensors to robots. Ensuring the security of these connected technologies is a key issue. Obviously, it should be kept in mind that the performance of a continuous biometric-based authentication system can also be affected by “temporary” factors such as excessive fatigue, a device other than the one commonly used, and time, which can impact the measurements of certain biometric traits.

It remains unclear in the literature which biometric traits work best when trying to deal with these circumstances that evolve over time versus behavioral patterns. Handling these situations therefore becomes critical if the goal is to create a robust and reliable system.

#### De-authentication

A direct consequence of continuous authentication is, of course, the possibility of de-authentication (Fig.  5d). This continuous authentication application subdomain is specifically concerned with detecting when a user whose identity has been previously evaluated leaves said device. Once the subject’s absence has been identified, all active secure sessions are terminated. Especially in a shared work environment, there is a risk of so-called Lunchtime Attacks occurring. In fact, when the worker whose identity is authenticated leaves their workstation, even if only momentarily, it potentially gives an attacker the opportunity to take control of it and be able to operate even with malicious intentions in the sessions inadvertently left active. Also in this case, if the goal is to avoid situations like the one just described, the eventual periodic re-authentication when inactivity times are recorded could in the long run annoy the user who maybe continues to stay on his workstation but is just thinking about how to best deal with the task. Imagine the boredom especially when this re-verification is related to password entry or when the biometrics involved requires a cooperative attitude from the user.

For these reasons, in Kaczmarek et al. (2018) the authors exploited and studied the potential of posture patterns of the user sitting in a classic office chair. One of the main advantages of using such biometrics is certainly the possibility of detecting it without any effort on the part of the user, in fact, his active collaboration is not required for its acquisition. A total of 30 subjects were recruited mainly from graduate students of a large public university. Data were collected in the form of 1.200 time series of samples reflecting the force exerted on each of 16 pressure sensors placed on a seat, acquired every 0.5 s for a 10-minute session, for a total of 19.200 samples per subject, per session. Two experiments were conducted, one for identification and one for de-authentication. The Random Forest (RF) classifier for the former obtained, on average, a true positive rate of 91.0%, an average false positive rate of 0.33%, and an average false negative rate of 8.68%. Evaluating user data over multiple sessions, on the other hand, the RF classifier produced only a true positive rate of 22%, a false positive rate of 5.2% and a false negative rate of 72.7%. The authors particularly pointed out that this biometric trait is of interest with regard to the vulnerability property. Indeed, in situations where the adversary knew the victim’s habits and schedule and had physical access to his or her workplace, the false positive rate for all subjects was just 0.33%.

### Identification

#### Facial dynamics

In the context of Industry 4.0, reliable recognition of the worker’s identity also benefits the production process. In fact, after the identity check, not only is the process more secure, but it is also possible to verify whether the subject is where he or she should be.

To face this kind of challenge, both in terms of identification but also authentication, the authors in Castiglione et al. (2020) analyzed a dynamic approach with respect to the classical static representations of the face by exploiting the dynamics associated with language. The experiments were conducted on a customized database containing short video clips capturing 48 subjects speaking short sentences. Features were extracted through a local spatio-temporal descriptor. The percentage obtained for the authentication task by pronouncing one-third of the passphrase was 89.81%, pronouncing two-thirds of the passphrase 97.79%, complete, however, 99.49%. For identification, on the other hand, with respect to the 3 different designated experimentation, accuracy rates above 98% were always achieved. The liveness of the acquisition allows also to fight most of the presentation attack strategies the obvious complexity of faking a time-dependent descriptor. In fact, a hypothetical impostor should be able not only to imitate the appearance of the unsuspecting user but also to simulate the behavioral dynamics when pronouncing a sentence. In an industrial application context, it is also necessary to have a system that minimizes the false rejection rate so as to avoid costly operational delays and ensure fast access at all times.

Therefore, the authors designed their system by trying to balance the sensitivity threshold between reducing the false rejection rate and the false acceptance rate. To ensure adequate recognition accuracy and reliability, the idea behind the architecture was to exploit a multilevel IIoT network approach. In the first layer, the feature acquisition was done through edge devices such as smartphones, industrial tablets, PCs, with the only hardware requirement to be equipped with a camera. The required computational resources, necessary for preliminary processing of facial movements around the mouth region, were limited. Instead, the extraction of the vector of local spatio-temporal features took place at the fog level. In the last level, the cloud one, a feed-forward deep neural network was used to obtain the match between identities. The experimentation took place in an IIoT environment of assembly and repair of systems for aerospace and defense applications.

#### Speech

Speech recognition systems represent a trend that became more entrenched during the COVID-19 pandemic (Balasco et al. 2021; Mumtaz et al. 2021). Just as people forced to work from home have found it increasingly functional to be able to control their appliances with their voice, industries may find it functional and safer to minimize more traditional manual input methods (such as keyboards or mice). An investment by organizations in such systems is both more inclusive for those employees who have physical or neuromotor disabilities and safer if one also reflects on the anti-contagiousness policies put in place during the COVID-19 pandemic that called for the periodic sanitization of all equipment with which one might come into contact. In industry, automated voice recognition systems could also enable workers to perform certain tasks without special effort and even faster and more easily.

Voice input allows for a simplification of communication between the machine and the user. Investing in this type of system has the advantage of giving workers the ability to be multitasking, using their hands for those functions that require them in a necessary way and delegating other tasks to the machine through voice. Therefore, with a view to adopting this type of system in an increasingly pervasive manner in the industrial context, there also arises the need to have adequate recognition accuracy for the person using it.

An application of speaker identification in the automobile industry was proposed in the paper of Srivastava et al. (2019). The work was separated into two categories. Using a database of isolated Hindi digits and Hindi sentences to train and test a system for multiple users, the first task was speaker identification. For speaker identification, a novel algorithm was employed that combined the advantages of the LPC and MFCC feature extraction techniques and made inferences with the Gaussian mixture model (GMM). The newly proposed method improved upon conventional MFCC features by 2.05% for isolated Hindi digits and by 12.41% for Hindi sentences. It also demonstrated an improvement of 53.26% over LPC for Hindi sentences and 32.51% over LPC for isolated Hindi digits. Adding speech and F16 noise to voice samples with varying degrees of distortion ranging from 0 to 20 dB was used to test the proposed features in a real-time noisy environment achieving improvements at all SNR levels.

#### Multimodal strategy

In the realm of biometric-based recognition systems, the use of a single biometric source can suffer from various limitations, such as sensitivity to environmental factors and susceptibility to spoofing attacks. To overcome these challenges and achieve a more robust and reliable authentication system, a multimodal approach that combines multiple biometric sources has been widely adopted. The multimodal strategy can improve the model’s generalization and accuracy by using a pool of inputs. Also, by combining more data, the system can make up for missing information from a single trait. This makes the recognition system more accurate and reliable. In the context of the IIoT, electrocardiogram (ECG) data has emerged as a popular biometric due to its high accuracy under ideal conditions  (Gokulkumari 2020). However, in real-world scenarios, ECG data can be corrupted by various types of noise and interference  (Venton et al. 2021), which can adversely affect the accuracy and reliability of the system. To deal with these problems and make the system more secure and reliable against spoofing attacks, a multimodal approach has been proposed that uses ECG data along with other biometric sources. In a recent study (Alkeem et al. (2021)), the authors fused ECG data with fingerprint and face information for gender recognition, identification, and authentication tasks. A ResNet50 model was used for the extraction of facial features and fingerprints, and a CNN model was used for ECGs. The fusion strategies included both feature and score fusion. The goal of the experiments was to find out how the fusion strategy, individual biometrics, and noise affect each other. In particular, the study investigated the impact of extreme noise on the performance of the multimodal approach, which is crucial for real-world applications in the IIoT. When using the feature fusion, the accuracy of user identification was 98.97% and the accuracy of gender classification was 96.55%. When using the score fusion, the authors found that the sum rule yielded an accuracy of 98.85% and 99.42% for the same task, respectively. The results clearly demonstrated that the multimodal approach significantly improved the robustness of the model to noise and enhanced its accuracy and reliability in countering the problem of false attacks as compared to the unimodal approach. These findings suggest that a system that integrates multiple biometric sources can compensate for missing information from a single trait and can achieve a more robust and accurate recognition system.

**Table 3:** Summary table for the area of workplace health monitoring

Paper	Aim	Biometrics	Dataset	Method	Performance
Soto et al. (2021)	Awakeness Focus Stress	Blood pulse wave Energy expenditure Galvanic skin response Heart Rate Heart rate variability Oxygen saturation Respiration rate Skin temperature	Private dataset: 14 subjects	Statistical analysis for feature extraction ML algorithms	*Awakeness* Accuracy: 80.4% Precision: 26.3% Recall: 24% *Focus* Accuracy: 67% Precision: 31.3% Recall: 19.2% *Stress* Accuracy: 76.8% Precision: 32.2% Recall: 26.1%
Rosemberg et al. (2019)	Work and non-work stressors Health outcomes	BMI Diastolic blood pressure Heart rate Systolic blood pressure Waist and hip ratio	Private dataset: 14 subjects	Statistical analysis	For ALI use of the clinical cut-off points resulted in more participants placed within the risk thresholds of waist/hip ratio and BMI
Dacunhasilva et al. (2021)	Stress	Keystroke dynamics Mouse trajectories	Private dataset: 25 subjects	Linear discriminant analysis ML algorithms	*Keystroke* Accuracy: 74.5% *Mouse* Accuracy: 73.3%
Ramírez-Moreno et al. (2021)	Mental fatigue	EEG Electrodermal activity Photopletismography Skin temperature	Private dataset: 17 subjects	Correlational analyses Multiple linear regression models	Accuracy: 88%
Ulinskas et al. (2018)	Mental fatigue	Keystroke dynamics	Private dataset: 4 subjects	Generalized linear discriminant analysis based on trace ratio criterion algorithm ML algorithms	Accuracy: 98.11%
Zhang et al. (2021)	Health monitoring diagnostic	Pressure distributions	Private dataset: 6 subjects 4 different types of stools and stools–amounts	DL algorithms	*Identification* Accuracy: 97.14% *Stool* Accuracy: 97.50% *Stools’ amount* Accuracy: 91.15%

Each research paper is reported according to the biometrics analyzed, the data sample used, the salient strategies, and the most interesting results obtained

## Health monitoring

Nowadays, more and more public and private entities are focusing on the concept of workplace health promotion (WHP), as it is clear that productivity is closely related not only to the qualification of the workforce but also to its well-being. A central role in this WHP paradigm is played by all those measures that prevent the accumulation of health risks among the working-age population.

**Fig. 6:**
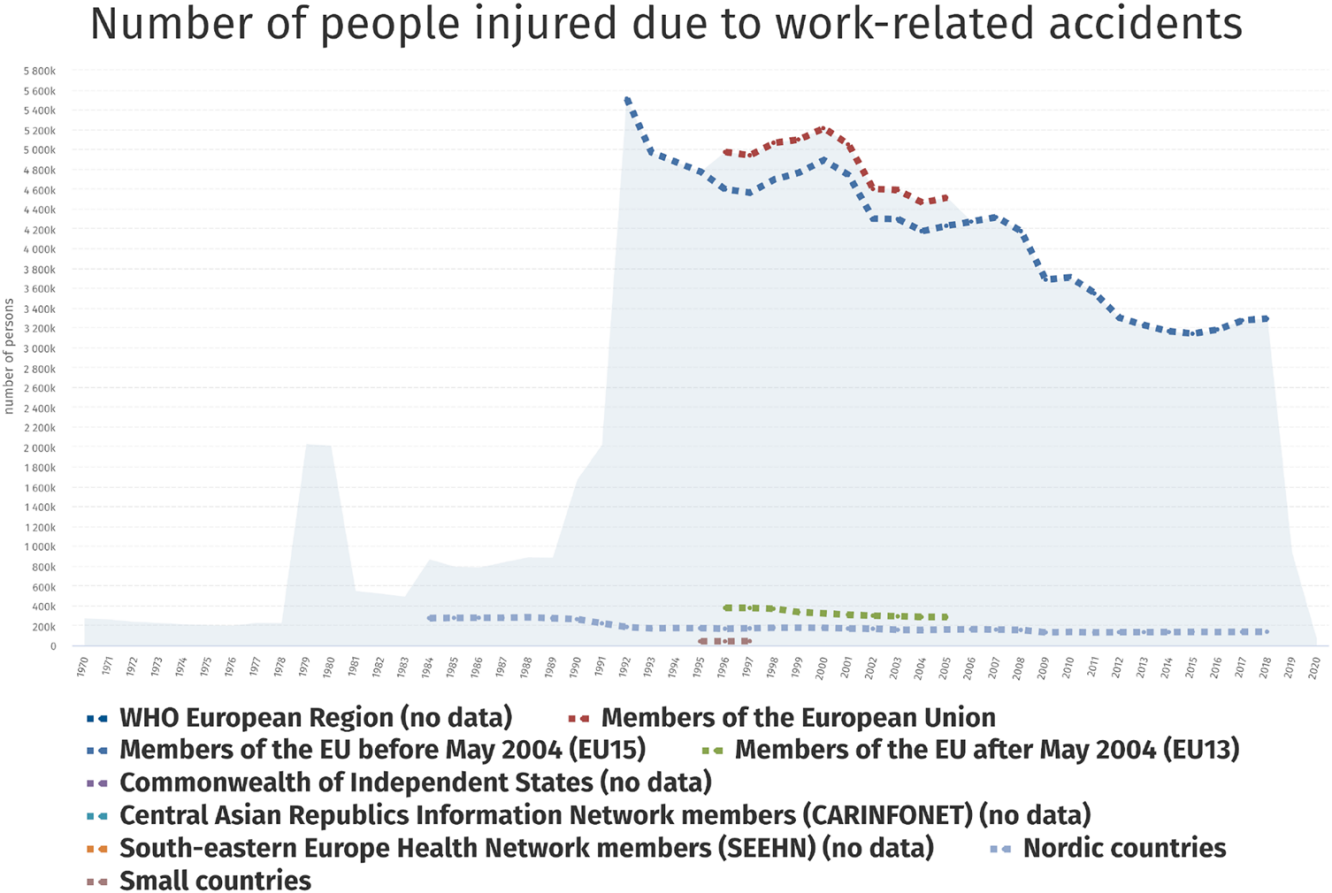
Number of people injured due to work-related accidents. Data from WHO Regional Office for Europe (https://gateway.euro.who.int)

Prevention of factors such as anxiety, depression, stress, and fatigue can also be cost-effective for several organizations to combat occupational injuries (Fig. 6), low productivity, and absenteeism. WHP can therefore, over time, allow for a financial return on the initial investment. For these reasons, having a tool to aid in the prevention and detection of these emotional and cognitive conditions becomes central.

Biometric screening is in this sense a now-popular measure for analyzing the health status of workers. In the workplace, various physiological characteristics (e.g., systolic blood pressure, diastolic blood pressure) can indeed be collected and exploited to ascertain the health status of individuals (Äikäs et al. 2020). Behavioral characteristics, on the other hand, may prove to be an interesting approach to identify the cognitive states in which the subject is in (Bisogni et al. 2022). Table 3 contains the most important details regarding the papers analysed in this section.

### Stress, focus, and awakeness

Work-related stress is the worker’s perception of not being able to complete assigned tasks, thus failing to meet expectations. If this condition persists and is experienced in a state of prolonged tension (allostatic load), it can be harmful and result in a real pathology, among the main ones that affect work activities. Its prevention is essential for organizations because it is one of the main causes of absenteeism and is also linked to a very high cost in terms of lost productivity (Concheiro-Moscoso et al. 2021). Stress can in fact lead to states of anxiety, depression, malaise and inattention with a consequent increase in the incidence of accidents.

Another cause of accidents is lack of alertness due to excessive sleepiness. Sleep deprivation is a condition associated with cognitive impairment affecting everything from memory to reaction time. In an occupational setting, it has a major impact not only on safety but also on the quality of interpersonal relationships among group members. Without adequate rest, people’s moods change and they become easily irritable and nervous. For this reason, several works have focused on detecting these conditions in order to prevent such situations.

Given the need to investigate the effects of emotional and cognitive state at work, Soto et al. (2021), in their paper, analyzed the possibility of exploiting biometric information to understand if a given subject at a certain time was awake, focused or was experiencing a sense of stress. Specifically, the authors through different biometric aspects in a real environment analyzed the behavior of knowledge workers by predicting in real time their level of stress, focus and sleepiness. The subjects participating in the experiment worked for the same company and performed tasks for a research group. The hardware used collected biometric signals related to heart, respiration and skin with low invasiveness. For each signal different characteristics were extracted and analyzed. In fact, it is clear the advantage of having multiple biometric streams that can be exploited simultaneously to obtain a correct detection. Among the various data collected (on eight-week with 14 office workers) for each condition analyzed, it was observed that for example heart rate variability and skin temperature were generally among those that give a greater contribution in making a decision than the three cognitive states and, in particular, for the detection of stress. Blood pulse wave instead turns out to be a good indicator for both focus and sleepiness but less so for stress. Respiratory rate does not turn out to be an interesting feature to analyze for awakeness but on the contrary for stress and focus. In general being the problem complex and difficult to solve, the results of accuracy and recall reported are overall low.

The difficulty of the problem is also evident in the work of Rosemberg et al. (2019). Here, the authors aimed to assess associations between work and non-work stressors, allostatic load, and overall health status. The study was conducted in a real-world setting among 49 female hotel housekeepers. Among the various parameters extracted for assessment, cardiobiometric measures such as systolic blood pressure, diastolic blood pressure, and heart rate once again appeared. This shows how well established this biometric trait is in the literature for this type of problem. Reports of high job strain and everyday discrimination were significantly associated with a high allostatic load index (ALI) quartile score (r = 0.39, p = 0.011; r = 0.41, p = 0.004, respectively). Workplace stress and everyday discrimination had moderate to substantial effects on ALI quartile scores. High ALI quartile score was significantly associated with at least one chronic disease (r = 0.40, p = 0.005), and it had a substantial effect size on chronic diseases. Because perceived stressors are multiple, it is difficult to capture them completely in their entirety and determine how and when they are triggered to affect worker health, so this work also highlights the difficulty of intercepting this condition in a real-life context where several factors, including strictly personal ones, come into play. The study conducted by Dacunhasilva et al. (2021) aimed to detect stress in knowledge work by analyzing information extractable from typing and mouse usage, while also evaluating the additional contribution of pressure data. To do this, low-cost external sensors were attached to computer peripherals that are already common in modern workplaces. In the process of getting the data, controlled stressors like the Stroop effect and mental math were used, and the results were checked with self-report measures. The study spanned several days and consisted of four sessions for each participant. Subject-independent classification rates of 74% with the keyboard device and 73% with the mouse device were obtained, showcasing the efficacy of the proposed approach. Notably, the addition of pressure information to the set of keys led to an average absolute improvement of 6% and 3% for the keyboard and mouse devices, respectively. These findings highlight the potential of the proposed approach for detecting stress in real-world scenarios and its practical applications in knowledge work environments.

### Mental fatigue

Mental fatigue occurs when you feel exhausted, overly tired and lacking energy. This condition can be caused by prolonged cognitive activity, but also by the constant decision-making that may be required of skilled workers in an industry, for example. For these reasons, being able to detect it can be critical in many work environments. There are several works in the literature that analyze mental fatigue (Ramírez-Moreno et al. 2021; Heaton et al. 2020). Among the main features most used and considered most appropriate for the detection of this condition is the monitoring of changes in neural activity through electroencephalography. This is accompanied by heart rate variability. Analysis of cardiac signals allows observation of the relationship between sympathetic and parasympathetic activity. Another indicator of emotional states is also electrodermal activity, which is used to predict changes in performance associated with cognitive fatigue and is generally a good indicator of sympathetic nervous system activity. An additional physiological measure whose use is well established in the state of the art for this purpose also appears to be body temperature.

A paper that takes advantage of the aforementioned characteristics while developing a system capable of being implemented also in different work scenarios, is that of Ramírez-Moreno et al. (2021). The features were extracted simultaneously through highly portable wearable devices, also applicable in industrial or real-life environments in general. The purpose was to detect mental fatigue condition, electroencephalography, photoplethysmography, electrodermal activity and skin temperature were the biometric information extracted from 17 subjects who took part in the experiment. The work was particularly interesting because the experimentation was very close to a real-world environment. The protocol can be safely applied during a work shift. Such an analysis could also be very useful to a company from the point of view of shift organization. In fact, through this study, one could also identify those tasks or shifts that contribute most to the onset of mental fatigue and based on this organize the work accordingly. Among the various features analyzed, noteworthy are the power ratios between the high and low frequency bands that contribute greatly to the 88% accuracy achieved.

Keystroke Dynamics is one of the least frequent biometrics or in any case, the use of which has decreased over the years, for the detection of this condition. One of the latest works that uses it for this purpose is that of Ulinskas et al. (2018). The authors in this work targeted to evaluate and recognize the daytime fatigue in a working day. The performances obtained seemed interesting (98.11% when only three qualitative classes of daytime fatigue, i.e. low, medium and high, are considered) but the work analyzed a small sample of subjects (only 4) and the evaluation of fatigue was made on shared but not certain considerations. In fact, the authors did not expressly ask the subjects for feedback on their level of fatigue, but they believed, empirically, that this was perceived particularly at the end of the working day. For these reasons, a more in-depth and targeted study would be opportune to evaluate its real effectiveness.

### Health monitoring diagnostic

Ensuring the health and safety of workers is not only necessary as far as reasonably possible but also beneficial. Monitoring the general health of employees can be particularly profitable for companies, as it can be a useful tool in combating productivity declines due to health problems and absenteeism due to illness. In particular, in those contexts where workers must manage and handle chemicals and hazardous substances, monitoring their health is an important part of the exposure assessment in the risk assessment paradigm. Therefore, for the realization of an intelligent work environment, one should not only invest in the integration of sensors and tools that support the various production and organizational phases, but also in systems that monitor the health status of workers. The substances from which to draw diagnostic information for continuous monitoring of health status can be various, urine, feces, body fat, but also heart-rate frequency and number of steps done (Cascone et al. 2021).

Based on these considerations, and since it is known that the information that can be extracted from excreted matter is particularly useful for health status assessment, in Zhang et al. (2021) the authors proposed an intelligent toilet system. One of the strengths of this solution, as presented, lay in its non-invasiveness, convenience for the user and respect for privacy as it did not integrate cameras or radio-frequency identification technologies. It was based on a system of 10 triboelectric sensors for recording the pressure variation on the toilet seat and a commercial image sensor for the analysis of excrement. The first sensors were used for the goal of having biometric identification (more than 90%), the second for health monitoring (recognition of simulated 4 different types of stools and stools’ amounts with accuracy more than 97% and 91%, respectively). The performance achieved on a sample of 6 subjects using a deep learning (DL) approach was promising for both goals. The data collected can also be uploaded on servers and shown to the workers involved to allow them to monitor their health status autonomously.

**Table 4:** Summary table for the area of quality work life

Paper	Aim	Biometrics	Dataset	Method	Results
Yu et al. (2017)	Impact on health outcomes	Blood pressure BMI Total cholesterol	Private dataset: 11.436 subjects 36.882 person-years	Statistical analysis	*Blood pressure* Not statistically significant impact *BMI* 1% change from baseline *Cholesterol* Not statistically significant impact
Song and Baicker (2019)	Impact on health outcomes	Blood glucose levels Blood pressure BMI Cholesterol levels	Private dataset: 32.974 subjects	Statistical analysis	No significant effects
Reif et al. (2020)	Impact on health outcomes	Blood pressure BMI Cholesterol level Glucose level Height Waist circumference Weight	Private dataset: 4.834 subjects	Statistical analysis	No significant effects
Bacevice and Ducao (2021)	Workspace quality	EEG Heart rate	Private dataset: a few tens of subjects	Statistical analysis	Higher median levels of delta and theta Lower median heart rate
Girardi et al. (2021)	Studying emotions and productivity	Galvanic skin response Heart measurements	Private dataset: 21 subjects	Qualitative analysis ML algorithms	Correlation between emotions and productivity *Positive vs negative* Accuracy: 79%
Züger et al. (2018)	Predict interruptibility	Heart rate Heart rate variability Keystroke dynamics Mouse interaction	Private dataset: 13 subjects	Statistical analysis ML algorithms	*Computer interaction* Accuracy: 74.8% *Biometrics* Accuracy: 68.3% *Fusion* Accuracy: 75.7%

Each research paper is reported according to the biometrics analyzed, the data sample used, the salient strategies, and the most interesting results obtained

## Quality work life

**Fig. 7:**
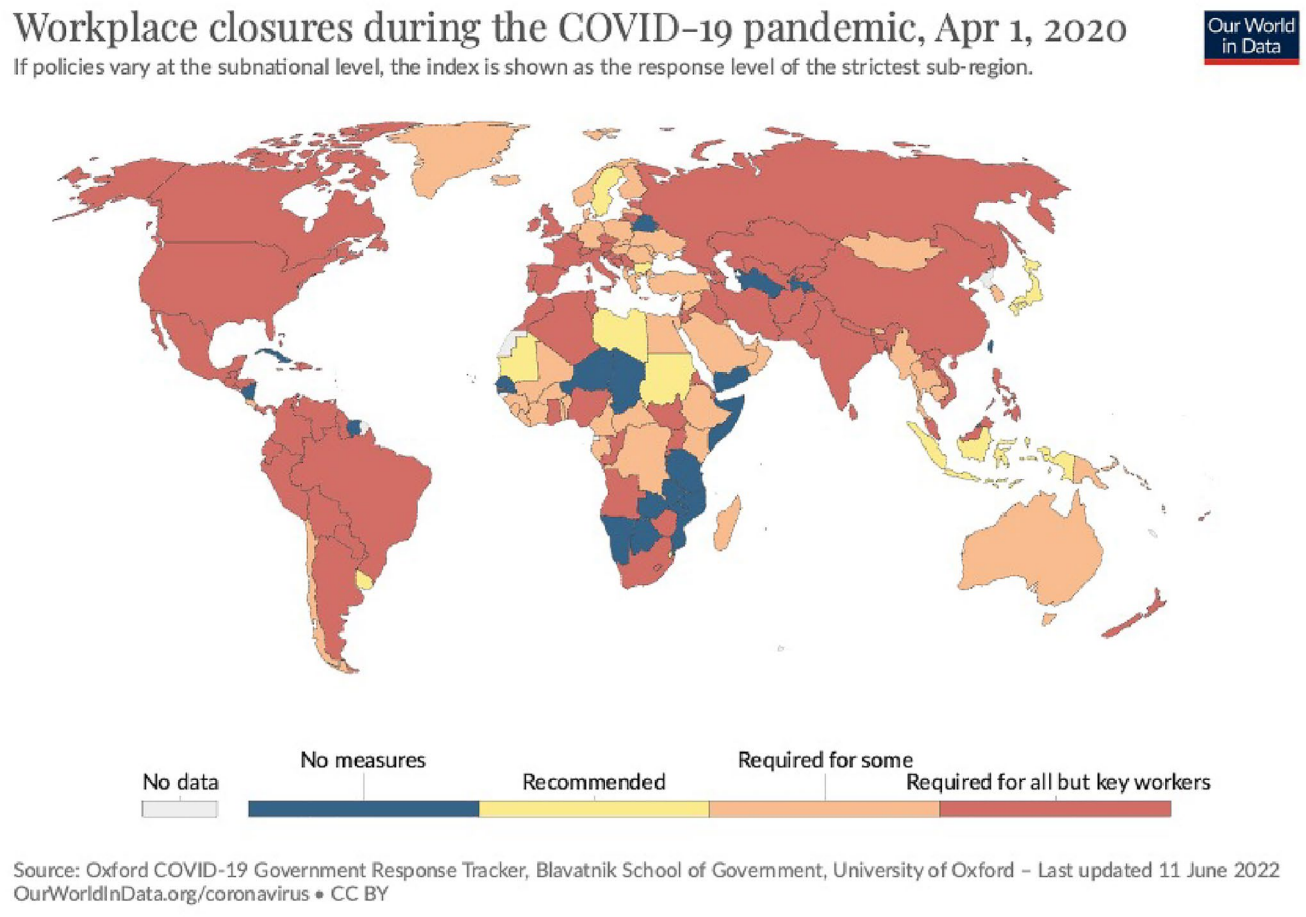
Workplace closures during the COVID-19 pandemic, April 1, 2020 (https://ourworldindata.org/covid-school-workplace-closures)

One of the foremost challenges confronting contemporary society are the repercussions of work-related stress. This condition is rooted in diverse factors, such as an excessive workload, pressing deadlines, an absence of control over one’s work, an unsupportive and contentious work milieu, and others. Recently, with the proliferation of remote work (a paradigmatic example of which is the fallout of the Covid-19 pandemic, Fig. 7), work-related stress has assumed a novel dimension. Despite remote work presenting various advantages, such as lower transportation costs and fatigue, reduced environmental pollution, and studies that attest to increased productivity in certain cases (Elshaiekh et al. 2018), many workers who operate from their homes or other off-site locations may feel increasingly isolated, experience a lack of communication, and encounter difficulty in maintaining a work-life balance. Furthermore, the absence of a structured routine and constant access to technological devices can exacerbate the stress of remote work. Consequently, it is imperative for both workers and employers to be cognizant of the hazards of work-related stress and to adopt effective strategies to prevent or manage it, whether in-person or in remote work contexts. Social policies aimed at safeguarding workers’ rights should ensure that individuals have sufficient freedom to achieve a healthy work-life balance. Work can have a profound impact on an individual’s life, often occupying their thoughts beyond the required working hours and influencing their psycho-emotional well-being. Dissatisfaction with one’s work life is a problem that can affect almost all workers at some point in their careers, regardless of their position or status. For these reasons, the study of the quality work life (QWL) has gained increasing attention in recent years. The term QWL refers to a dynamic, multidimensional construct that emphasizes the employee’s overall quality of life in the workplace. The objective of this approach is to influence the structural and managerial systems of organizations while taking into account the socio-psychological needs of workers. This generic term incorporates an individual’s feelings about their working conditions, recognizing the significant interdependence between environmental stimuli and physiological and cognitive responses (Persiani et al. 2021). By integrating biometric data into the study of QWL, researchers can gain a more comprehensive and objective understanding of the impact of work on an individual’s physical and emotional well-being. For example, if biometric data indicates that a worker is experiencing high levels of stress, interventions such as mindfulness training or regular breaks may be recommended. Additionally, biometric data can be used to evaluate the effectiveness of these interventions in real-time, allowing for continuous improvement and refinement. Table 4 contains the most important details regarding the papers analysed in this section.

### Biometric health outcomes

A healthy, fit worker means having higher productivity and lower healthcare costs. The wellness strategies put in place encourage a change in one’s lifestyle. Among the various activities supported are certainly those to keep physically active, as the positive effects of sport on an individual’s health are well known. If these are practised on a corporate level, social relationships and team cohesion also benefit. Wearable tracking devices are now common hardware, and with the increased development of mobile health applications, several employers are offering physical activity tracking applications.

Prompted by such observations, the authors in Yu et al. (2017) decided to evaluate the correlation between individuals’ participation in a workplace physical activity monitoring program and the change in several biometric measures such as body mass index (BMI), blood pressure but also total cholesterol. The research sample consisted of active workers and adult dependents who were continuously enrolled in a qualifying health plan and had undergone at least two biometric screenings (totalling 36.882 person-years and 11.436 unique individuals) from 2011 to 2014. The authors utilized difference-in-differences regression to calculate the impact of using a physical activity tracking app on three parameters. Results showed that individuals who participated in the app had a significant reduction of 0.275 in their BMI in the post-period, compared to the control group. This represents a 1% change from their initial BMI. However, the study did not have a statistically significant effect on cholesterol or blood pressure. Attention was also paid to better understanding the underlying reasons for any recorded effect. However, consistent with other and more recent work in the literature, the results obtained put a brake on the actual effectiveness of these programs on the biometrics under investigation.

Further confirmation comes from the study of Song and Baicker (2019). The study they suggested lasted 18 months and included a group of workers with medium and low incomes; clinical and biometric information analyzed include blood pressure, BMI, blood glucose and cholesterol levels. Just as expected, again the benefits of the program involved physical activity rate and weight management without affecting biometrics indicative of health status instead.

Even with 24-month studies, the results did not change (Reif et al. 2020). The biometrics involved that reflect and monitor health status are therefore a useful tool for assessing the effectiveness of such programs. On the basis of the measurements obtained, one can then also outline the company’s policy in this regard and decide, if so, whether or not it is worth investing in such an activity.

### Wellness and productivity

Cognitive and emotional states play a fundamental role in work performance, both in those tasks of responsibility where attention is required and in those activities whose main prerogative is creativity and originality. The work environment and, in general, its architecture have an impact on the behavior and emotional state of those who spend their time there.

In Bacevice and Ducao (2021), the authors therefore aimed to analyze how office architecture affected the neurophysiological responses of a few tens of individual workers. An experiment was undertaken in this study to investigate and assess how participants experienced their work space using biometric data such as heart rate monitors and portable EEG sensors. In particular, data were collected on some workers who moved from an old office to a new one that was more comfortable and suited to their needs. The results (higher median levels of delta and theta, lower median heart rate) showed how users perceived their new spaces in a more relaxed way. This then suggests the interesting use of biometrics as a monitor of the effects and impacts of the architecture and design of the work environment on the emotional and cognitive spheres of workers.

In general, emotional and cognitive alterations may be due to a condition of prolonged stress that can impact the occupational balance and routine of workers who experience it. Therefore, assessing stress not for clinical and medical purposes but to measure its impact on quality of life is the goal of Concheiro-Moscoso et al. (2021). The authors implemented a protocol for assessing the impact of work stress in the subjects’ daily lives by also basing the analysis on biometric parameters (such as heart rate) collected through inexpensive, wearable hardware devices. In addition to using suitable hardware to obtain records of information during daily activities, participants in the experiment recruited from the workplace should also be given a questionnaire to assess different aspects that may affect their quality work life.

The impact of emotions on job performance, on the other hand, is the subject of the work (Girardi et al. 2021). The authors in their study analyzed the emotions experienced by 21 software developers in the workplace. The information extracted was collected through self-diagnosis and biometric detection. The biometrics examined, including galvanic skin response and heart-related measures, were acquired with noninvasive sensors. The work showed the existence of a correlation between self-reported emotions and productivity. On the other hand, regarding the results on emotion classification (0.79% accuracy for the detection of positive and negative emotions), these were still far from being performant, worse than those obtained in a controlled context such as the laboratory. Negative emotions, such as frustration or nervousness, on the other hand, are often linked to all those events or setbacks that occur at inopportune times, forcing workers to stop what they are doing. The timing of the interruption, which is also often necessary to have a confrontation, has a great impact on overall productivity.

For these reasons, Züger et al. (2018) in their work analyzed the measure that indicated a subject’s willingness to be interrupted during a task. They conducted a 2-week field study with thirteen professional software developers to analyse various data extracted from their computer interaction and biometric sensors. The extracted data included, for example, heart rate, sleep, and physical activity measures. These were then combined through experience sampling with information gathered through a self-assessment of one’s interruptibility. Recognition of this propensity to be interrupted appears to achieve promising results when a combination of biometric data and data extracted from the subject’s interaction with the computer was used as input (75.7% accuracy). In general, however, using data from only the latter interaction seems to yield better results than using biometric data alone (74.8% vs. 68.3% accuracy). However, it would be interesting in such a study to consider different biometrics that perform better when it comes to intercepting states of concentration or distraction.

## Open issues and limitations

**Fig. 8:**
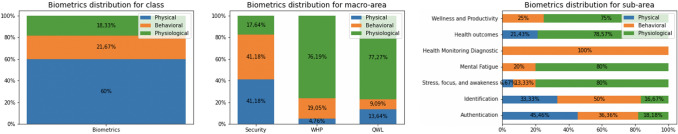
From left to right: percentage of use of physical, behavioral and physiological biometrics out of the total documents analyzed; percentage of use of physical, behavioral and physiological biometrics in the three macro-areas highlighted; percentage of use of physical, behavioral and physiological biometrics with respect to the sub-area of security, health monitoring, quality work life

Industry 4.0 is a rapidly evolving concept that combines several paradigms, including industrial IoT, Big Data, and cloud computing (Barroso et al. 2022). On the other hand, the use of biometrics has become so pervasive in many industries that it is fundamental to some aspects of our daily lives. It is clear from the above analysis that the integration of these concepts has been directly explored in only a few works. To achieve optimal integration and performance between these two paradigms, it is necessary to have full knowledge of the limitations and to resolve a number of open questions in the near future (Gao et al. 2022). From the perspective of potential application, these two concepts have been investigated in terms of physiological, behavioral and physical biometrics outlining three macro-areas of industrial applications: security, health monitoring and quality work life. Figure 8 summarises, for the selected papers, the distribution of physical, physiological, and behavioural biometrics in relation to these three macro-areas and the sub-categories associated with them. In general, for all 3 possible application scenarios, regardless of the biometric trait analyzed, there are shared issues. The main problem always concerns the issue of privacy: of how personal data is actually handled but also the perception of how it is done. For the first issue, workers may have fears and apprehensions that a malicious third party may intrude during the collection, transmission and storage of their personal data by stealing, altering or compromising it. In the works cited, none highlight this aspect, focusing mainly on the tasks only recognizing the subject or classifying his or her cognitive or emotional state. However, there are several works in the literature that from a theoretical point of view, without validating their method in a real situation, address this issue by proposing protocols for secure and private communication between the various edge devices involved  (Irshad et al. 2022; Wazid et al. 2020). The second issue concerns workers’ perceptions of how their information is handled and the pressure they may feel knowing they are constantly being monitored. This can lead to a sense of unease and frustration, which can cause people to change their behavior. An additional aspect that should not be underestimated is the possible evaluation that employers might make of the results obtained, which in any case have an element of error and uncertainty despite the fact that some biometric data are particularly good performers for certain tasks. In terms of implementation strategies, although the accuracy and consequently the effectiveness of deep learning methods can be quite high, their adoption in real-world industrial applications is often constrained by their computational requirements. Implementing Deep Learning models requires expensive hardware resources, such as powerful GPUs or cloud computing platforms. In addition, models may need to run in real time or near real time in industrial applications, making the computational load even more challenging. Implementing Deep Learning techniques for biometric recognition in real-world scenarios may therefore be difficult. One of the biggest limitations to the development of this study on biometrics and Industry 4.0 also relates to existing datasets that are mostly small or private, not allowing reproducibility and progress in the area. Overall, also not to be underestimated are a number of ethical issues associated with biometrics that are complex and multifaceted. Below is a more specific focus on the biometric groups under consideration.

### Physiological biometrics

For physiological biometrics, the main challenges related to the implementation of a biometric system based on these features are definitely related to the acquisition protocol, the effective ease of use of the hardware, and the reliability in the medium to long term of the extracted data. One of the main limitations in evaluating the effectiveness of these biometrics in the work environment is that most studies using such biometrics are not conducted in real-world situations, simulating the environment in the laboratory, e.g., using immersive virtual reality systems  (Higuera-Trujillo et al. 2020), or, in non-laboratory environments that only potentially replicate a work context  (Ramírez-Moreno et al. 2021; Libert and Van Hulle 2021). When the goal, on the other hand, is to intercept a certain emotional or cognitive state, there are additional critical issues that must be taken into account. The relationships between emotions and the signals that are recorded are very complex. Between the physiological responses that are observed and the psychological elements that cause them, there can be the following relationships: one-to-one, that is, a psychological condition is associated with a single physiological signal; one-to-many, when the psychological condition is manifested by several physiological signals; many-to-one, that is, several psychological conditions are associated with the same physiological signal; many-to-many, when several psychological conditions are manifested by several signals  (Lohani et al. 2019). Among these different scenarios, the one-to-one ones are certainly the most interesting to study for recognition purposes, but isolating them with certainty is still complicated. The results therefore that are obtained must be analyzed with caution.

*Industry context* Physiological biometrics, such as electrocardiogram analysis, may be more suitable for industries where continuous monitoring is needed, such as health care or financial services. In settings where high physical labor intensity is required, physiological biometrics may not be the best choice for user identification, as fatigue and stress can affect biometric parameters such as heart rate and brain waves, making detection accuracy difficult. Also, in some industries, the use of physiological biometrics may not be practical or hygienically acceptable because of the need for physical contact with the sensing devices.

*Ethical issues* The use of physiological biometrics could lead to discrimination based on health status or other personal characteristics. They may be able to reveal psychological states and traits or personality characteristics (extroversion, neuroticism, risk aversion, pessimism, tenacity, empathy...) (Gondesen et al. 2019). This could lead to exploitation or misuse of data and result in unfair treatment, limiting opportunities for certain individuals. Therefore, the protection of this personal information (beyond unauthorized access by third parties) raises non-trivial questions, for example, how to control which features are extracted (Bonaci et al. 2014).

### Physical biometrics

Physical biometric features such as the face, fingerprint or iris, although widely used because of their high discriminatory power, still present problems. Capturing certain traits can be particularly invasive, expensive to set up, or unsuitable in some work settings or for certain categories of people who may have difficulty using biometric systems because of health problems or disabilities. For example, if a person has a skin disease or hand injury that prevents fingerprints from being read. In addition, some physical biometrics are easily subject to change over time such as weight gain or loss, aging, or even the use of glasses, can affect the accuracy of biometric systems. This can lead to false positives or false negatives. The security aspect also should not be underestimated. These traits are easily counterfeited, replicated, or altered, thus putting the security of the system at risk.

*Industry context* Physical biometrics may not be as effective in industries where the workforce is primarily composed of black-collar workers, such as construction or manufacturing, as these types of work, for example, could result in hand injuries or facial alterations due to the use of protective equipment. Furthermore, in these industries where employees engage in high levels of physical exertion, such as manufacturing or agriculture, the use of physical biometrics may be impractical or inconvenient for workers as they may have to stop and interact with sensing devices frequently.

*Ethical issues* Some physical characteristics, may result in unfair discrimination among individuals, particularly if the biometric system was not designed with diverse populations in mind or if there are individuals with disabilities or physical deformities.

### Behavioral biometrics

The main problem with a system that relies only on behavioral traits is related to the degree of reliability. It is well known that behavioral biometrics is not stable over time and, indeed, is also influenced by the psychological condition of the individual. For example, an individual’s typing speed or mouse movements may vary depending on the level of fatigue or emotional state, which can lead to inaccuracies in identification and authentication. Unlike physical biometrics, which is based on static physical characteristics, behavioral biometrics requires a continuous flow of data over time. This means it may not be feasible or effective in situations where data collection is limited or sporadic.

*Industry context* Behavioral biometrics, such as gait analysis or keystroke dynamics, may be more suitable for industries where continuous monitoring is not necessary, such as online banking or e-commerce. In industries where employees frequently switch devices or work remotely, capturing and analyzing consistent behavioral data can be difficult. In addition, industries where employees are subject to high levels of stress or emotion, such as emergency services or social work, the use of behavioral biometrics may be less effective because emotions can affect behavior, making it difficult to establish reliable behavioral patterns.

*Ethical issues* Behavioral biometric systems may not always be transparent in terms of what data are collected and how they are used. Behavioral biometric data could be used for purposes other than identification, such as customized assessments to monitor employee performance.

## Future challenges

There are several challenges that must be addressed in order to ensure the effective and secure application of biometric technology in Industry 4.0 (Gao et al. 2022). Some of these include:

- Universality and fairness: biometric technology must work across a wide range of demographics, including different ethnicities, ages, genders and also vulnerable groups. This requires the development of biometric algorithms that are inclusive and unbiased, as well as the collection of diverse training data. Biometric technology must be used fairly and ethically, without discriminating against certain groups or individuals. This requires careful consideration of the social and ethical implications of biometric technology, as well as the development of appropriate governance frameworks and policies.
- Cost-effectiveness: biometric technology can be expensive to implement and maintain, and Industry 4.0 applications must ensure that the benefits of biometric systems outweigh the costs. This requires careful analysis of the costs and benefits of implementing biometric technology, as well as ongoing monitoring and optimization of systems to maximize their effectiveness and minimize costs.
- Hardware: biometric sensors must be of high quality and durability, as they are often used in harsh industrial environments. This requires the development of robust and reliable sensors that can withstand dust, moisture, and other environmental factors. In addition, biometric sensors often require significant amounts of power, which can be a challenge in remote or mobile applications. This requires the development of low-power sensors and power management systems that can extend the battery life of devices. Biometric sensors and hardware should also be designed to be as unobtrusive as possible, to avoid disrupting workers’ daily activities or causing discomfort or inconvenience. In addition, biometric technology should be easy to use, with clear instructions and user interfaces that do not require the involvement of third parties. Workers should be able to use the technology without significant training or assistance, and the technology should not add to their workload or create additional administrative burdens.
- Availability and quality of data: the availability and quality of data are critical factors in the effective application of biometric technology in Industry 4.0. Biometric systems rely on large amounts of high-quality data to accurately identify and authenticate individuals, and to enable real-time monitoring and analysis of worker activities. In almost all of the work considered, experimentation is first preceded by data acquisition, which then remains private. Therefore, the literature lacks a comprehensive and extensive data collection that allows exploratory investigations but focuses on the implementation of biometric systems and the analysis of their results only. We therefore report on the TILES-2018 (Tracking Individual performance with Sensors) public dataset presented in the article of Mundnich et al. (2020). Although only hospital staff were involved, there was intensive multi-modal, environmental (both workplace and home) and contextual (including through questionnaires) data collection in the study. The goal is to learn about the dynamic relationships between individuals, work behaviors, and intercept well-being in order to determine when it is perceived.

The main challenge facing the implementation of biometric systems in industrial environments is to develop a system that can achieve high performance in all three categories outlined. Some biometric modalities can serve multiple purposes, and it would be interesting to optimize their contribution by carefully analyzing their strengths and weaknesses. By maximizing the potential of biometric systems, industries could therefore simultaneously improve operational efficiency and promote employee welfare.

## Summary and conclusions

Industry 4.0 with its trend towards automation enables real-time communication between cyber-physical systems and with workers in charge of various tasks. The human being thus becomes a fulcrum in this system, as it is up to the operator to understand, control and supervise the automated processes. It is necessary to develop interfaces and services that allow the user to have this interaction in a simple and immediate way, but at the same time guarantee a high degree of protection of the data the person accesses, ensuring that only authorized personnel can view it.

The workplace is not just a symbol of one’s professional identity, understanding how individuals cope with work tasks and their overall well-being, including with respect to the environment in which they spend their working day, can be useful in then applying just-in-time corrective solutions. Being constantly under pressure can manifest in brain fatigue resulting in loss of clarity. This can be a problem in some jobs and be the cause of many accidents in which both economic and, unfortunately, human losses occur.

This survey paper has reviewed recent research on the use of biometric techniques in Industry 4.0 that aims to address these issues. From this analysis three macro research groups emerge, that of security, analysis of the quality of working life and health monitoring. After highlighting for each category the most used biometrics and the best strategies implemented for their use, the main contribution of this state of the art analysis paper is that it highlights for this new and growing paradigm the limitations, challenges and prospective research opportunities to help future researchers in the field in the transformation of the current Industry to 4.0 one.

## Data Availability

No data available.

## Footnotes

1 https://www.vosviewer.com.

2 https://www.scopus.com.

3 https://dl.acm.org.

4 https://ieeexplore.ieee.org.

5 https://www.scimagojr.com.

6 http://scie.lcc.uma.es.
